# The Prognostic Value of *p16* Hypermethylation in Cancer: A Meta-Analysis

**DOI:** 10.1371/journal.pone.0066587

**Published:** 2013-06-21

**Authors:** Xiang-Bin Xing, Wei-Bin Cai, Liang Luo, Long-Shan Liu, Hui-Juan Shi, Min-Hu Chen

**Affiliations:** 1 Department of Gastroenterology, The First Affiliated Hospital of Sun Yat-sen University, Guangzhou, China; 2 Department of Biochemistry, Zhongshan Medical School, Sun Yat-sen University, Guangzhou, China; 3 Laboratory of General Surgery, The First Affiliated Hospital of Sun Yat-sen University, Guangzhou, China; CEA - Institut de Genomique, France

## Abstract

**Background:**

The prognostic value of *p16* promoter hypermethylation in cancers has been evaluated for several years while the results remain controversial. We thus performed a systematic review and meta-analysis of studies assessing the impact of *p16* methylation on overall survival (OS) and disease-free survival (DFS) to clarify this issue.

**Methods:**

We searched Pubmed, Embase and ISI web of knowledge to identify studies on the prognostic impact of *p16* hypermethylation in cancers. A total of 6589 patients from 45 eligible studies were included in the analysis. Pooled hazard ratios (HRs) with 95% confidence interval (95% CI) were calculated to estimate the effect using random-effects model.

**Results:**

The analysis indicated that *p16* hypermethylation had significant association with poor OS of non-small cell lung cancer (NSCLC) (HR 1.74, 95% CI: 1.36–2.22) and colorectal cancer (CRC) (HR 1.80; 95% CI 1.27–2.55). Moreover, the significant correlation was present between *p16* hypermethylation and DFS of NSCLC (HR 2.04, 95% CI: 1.19–3.50) and head and neck cancer (HR 2.24, 95% CI: 1.35–3.73). Additionally, in the analysis of the studies following REMARK guidelines more rigorously, *p16* hypermethylation had unfavorable impact on OS of NSCLC (HR 1.79, 95% CI: 1.35–2.39) and CRC (HR 1.96, 1.16–3.34), and on DFS of NSCLC (HR 2.12, 95% CI: 1.21–3.72) and head and neck cancer (HR 2.24, 95% CI: 1.35–3.73).

**Conclusions:**

*p16* hypermethylation might be a predictive factor of poor prognosis in some surgically treated cancers, particularly in NSCLC.

## Introduction

Despite the recent reduction in incidence and mortality, cancer is still a worldwide health burden and leads to more deaths than heart disease in some regions [1]. Surgical resection can be performed to remove the tumor if neither lymph node nor distant metastasis were present, while recurrence rate after surgery remains high [2], [3]. Moreover, numerous cancers at the time of diagnosis are at advanced stage and the treatment options are limited, resulting in the persistent high mortality of cancers.

A lot of efforts have been made to investigate the prognostic biomarkers including epigenetic markers in cancers, helping to identify high-risk cancer patients who might need adjuvant treatment after surgery. *p16*, located on chromosome 9p21, is one of the most commonly altered genes in human cancers and functions as an important tumor suppressor [4], [5]. Promoter hypermethylation of *p16*, leading to the loss of *p16* activity and tumor progression, is a frequent epigenetic event in various cancers [6], [7]. Although the impact of *p16* hypermethylation on prognosis of patients with cancer has been explored recently, the prognostic value of *p16* hypermethylation in different tumor types remains conflicting because heterogeneous results were reported in studies and some of them included a small number of patients. To elucidate this issue, we performed this systematic review and meta-analysis to assess the prognostic significance of *p16* hypermethylation in various types of cancer.

## Materials and Methods

### Search Strategy and Selection Criteria

We searched Pubmed, Embase and ISI web of knowledge to identify studies that assessed the prognostic value of *p16* hypermethylation in patients with carcinomas who underwent surgical resection of a tumor. The search strategy was the following terms:“*p16*”, “*CDKN2A*”, “methylation”, “cancer”, “carcinoma”, “prognosis”, “prognostic”, and “survival”. The search ended in Oct, 2012, and no lower date limit was applied. References cited in selected articles were also searched manually to identify other relevant studies. Although our search did not have language limits initially, for the full-text reading and final evaluation we only performed the review of the studies published in English language. Conference abstracts were not selected for our analysis due to the insufficient data reported in them.

Criteria that an eligible study has to meet were as follows: (a) to evaluate the relationship between *p16* methylation and overall survival (OS) or disease-free survival (DFS) of patients with carcinoma; (b) to assess *p16* methylation status using methylation-specific polymerase chain reaction (MSP) or quantitative MSP (qMSP); (c) to determine *p16* methylation in surgically resected primary tumor tissues (not in normal tissues or in body fluids such as blood and sputum) (d) to possess a study sample size greater than 20.

Two reviewers (XXB and CWB) independently judged if studies screened were eligible. Disagreements were resolved by discussion. If the results reported in identified studies have the possible overlap (e.g., same authors, institutions), only the most informative study was involved in the analysis.

### Data Extraction and Management

Two authors (XXB and CWB) independently reviewed each eligible study and extracted data. The database recorded the most relevant data encompassing author’s name, year of publication, region, tumor type, stage of disease, number of patients, methylation rate, methylation detection method and follow up.

### Methodological Assessment

For the methodological evaluation of the studies, three investigators (XXB, CWB and CMH) read through each publication independently, and assessed and scored them according to REMARK guidelines and ELCWP quality scale [8], [9]. The three readers provided the quality scores and compared them, and then reach a consensus value for each item.

The REMARK guidelines include the details on 20 items, allowing for the evaluation of the studies by study purpose, study design, patient inclusion, biomarker detection, statistical analysis methods, report of results, etc [8]. While the ELCWP quality scale system examined several aspects of methodology, which fall into four major groups: the scientific design, laboratory methodology, the generalisability of results and the analysis of the study data. Each category yielded a maximum score of 10 points, and therefore 40 points are the total maximum theoretical score. If a certain item was not suitable in one study, its value was not taken into account in the total of the related category. We gave the total score using percentages, with a range of 0–100%, and a higher score represented a better methodological quality [9].

### Statistical Analysis

To aggregate the survival data quantitatively, the impact of *p16* hypermethylation on OS or DFS was measured by hazard ratio (HR). Studies providing univariate or multivariate analysis results for survival were used for the aggregation of the survival data. HRs and their corresponding SEs were directly extracted from studies or estimated using an approach reported in a previous publication [10]. The most accurate approach is to retrieve the HR estimate and 95% confidence interval (CI) directly from the publication, or calculating them using the parameters reported in the manuscript: the O-E statistic and variance. Otherwise, the number of patients at risk in each group, the number of events and the log-rank statistic or its p-value were obtained to allow for an approximation of the HR estimate and its variance. If the study did not report a HR but gave the data in the form of the survival curve, survival rates at certain specified times were extracted from them for the reconstruction of the HR estimate and its SEs, with the assumption that the rate of patients censored was constant during the follow-up.

The extracted individual HR estimates were combined into a summary HR using the method reported previously [11], which comprises the application of a fixed-effects model with the assumption of the homogeneity of the individual HRs. We evaluated statistical inter-study heterogeneity using I^2^ statistics. If the assumption of homogeneity had to be rejected, a random-effects model was applied in a subsequent stage. An observed HR >1 implied a worse survival for the group with *p16* hypermethylation and would be considered to be statistically significant if the 95% CI for the overall HR did not overlap 1.

Extracted HRs were pooled using the revman systematic review and meta-analysis software package (Review Manager Version 5.1). Publication bias was assessed by using a method reported by Egger et al [12]. We also explored reasons for statistical heterogeneity using meta-regression, subgroup analysis and sensitivity analysis. The analysis of meta-regression and publication bias was performed by using STATA version 10.0.

## Results

### Study Selection and Characteristics

**Figure 1:**
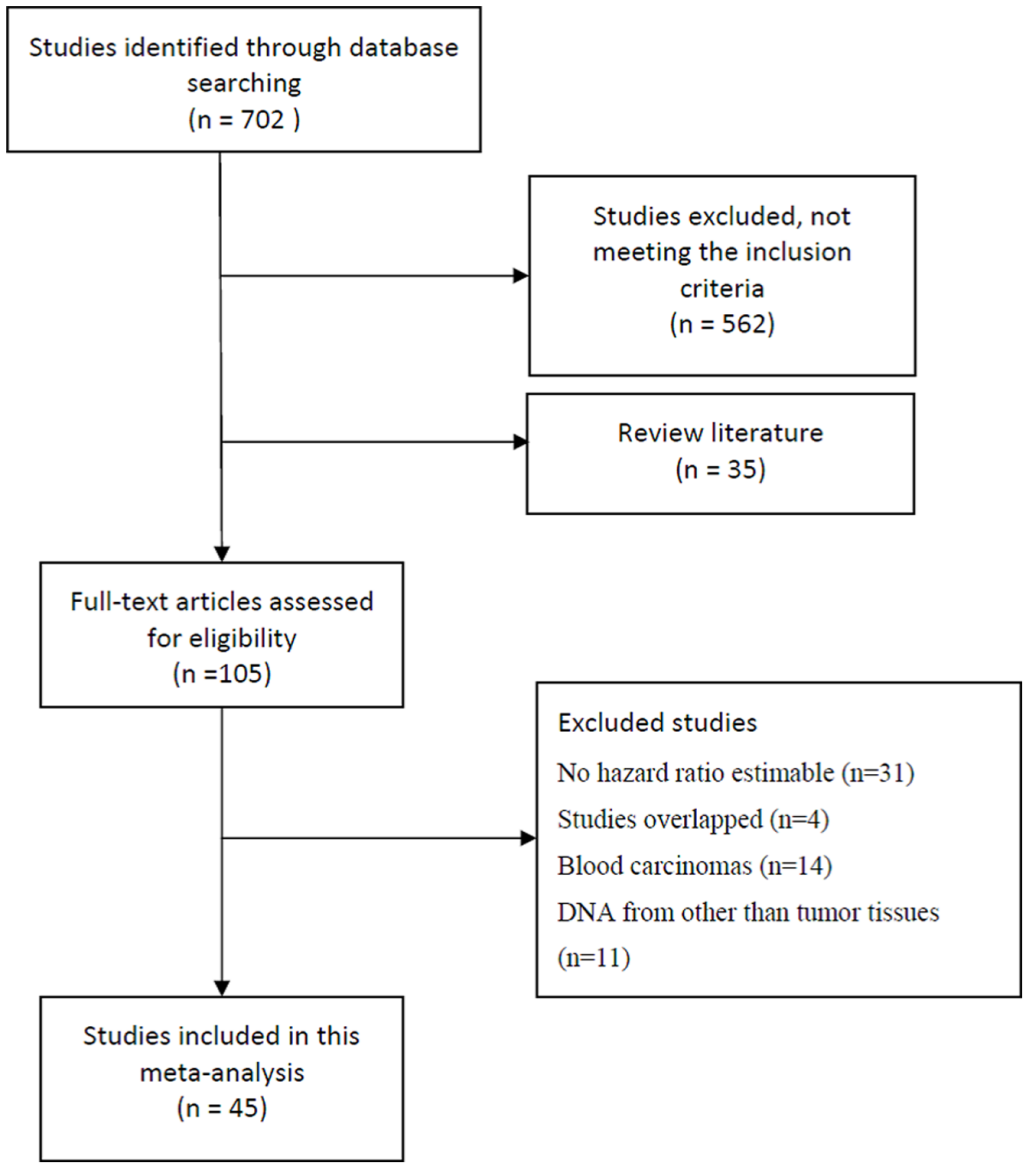
Flow chart of study inclusion.

In total, 702 articles were identified from a literature search using the search strategy above-mentioned [Figure 1]. Of these, 105 studies assessing the prognostic value of *p16* hypermethylation in patients with cancer were considered for detailed review. Upon further evaluation, 11 were excluded because the authors assessed *p16* methylation using DNA from other than tumor tissues, 31 excluded because it could not permit the calculation of HR estimate owing to the insufficient data reported in these studies, 4 excluded because the data reported in them were overlapped with other studies, and 14 excluded because the assessment was conducted on blood carcinomas. As a result, 45 publications were finally enrolled for analysis of the prognostic value of *p16* hypermethylation in carcinomas [13]–[57] [Figure 1].

The variables extracted from these 45 studies eligible for the meta-analysis are summarized in table S1. All eligible studies were published between 1999 and 2012. Fifteen studies evaluated patients from Japan, 9 from China, 7 from USA, 6 from Spain, the remaining 8 from multiple countries. The included studies comprised 6589 patients, with sample sizes ranging from 24 to 902 patients (mean 146). Twenty-four of these studies enrolled less than 100 patients and 10 studies included more than 200 patients (table S1). Multivariate survival data were available in 10 studies (22.2%). *p16* gene was found to be methylated in 33.6% of patients. A significant association between *p16* hypermethylation and the survival outcome of cancer patients was recorded in 41.5% of studies on OS, and 35% of studies on DFS.

We calculated the individual HRs of the 45 included studies using one of the three methods mentioned in the Materials and methods section. Fifteen studies provided the data from which we can directly obtain their HRs. In 10 studies, HRs were approximated by the total number of events and the log-rank statistic or its p-value. While for the 20 remaining studies, we had to extrapolate HRs from the graphical representations of the survival distributions.

### Association of*p16* Hypermethylation and Survival of Non-small Cell Lung Cancer (NSCLC) and Colorectal Cancer (CRC)

**Figure 2:**
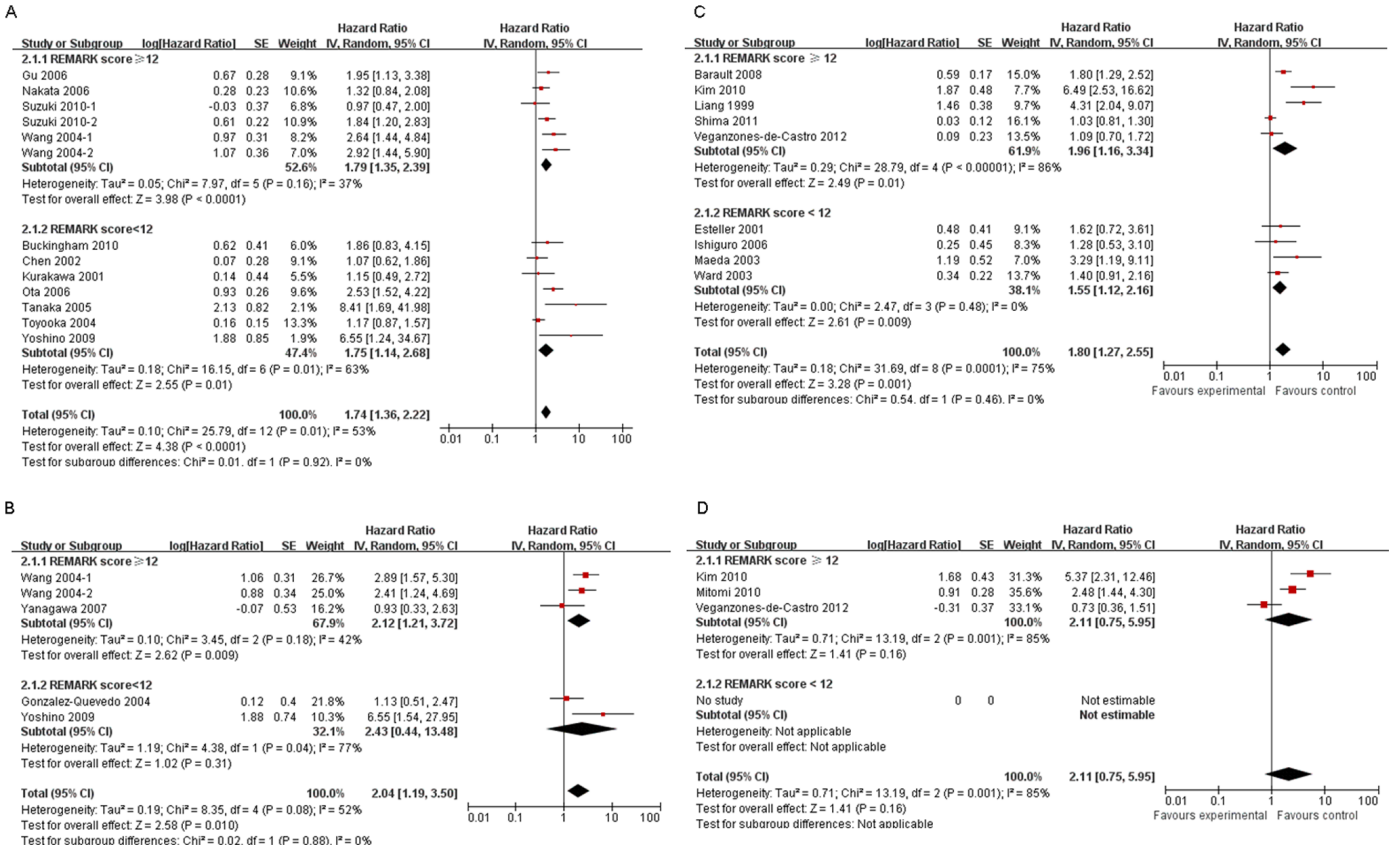
Meta-analysis of the effects of*p16* hypermethylation on OS (A) and DFS (B) of NSCLC, and on OS (C) and DFS (D) of CRC. Subgroup analysis by REMARK score is also demonstrated. Results are presented as individual and pooled hazard ratio (HR), and 95% confidence interval (CI).

In NSCLC, the pooled analysis of overall survival (OS) was based on 11 publications including 1654 patients. A significant association of *p16* hypermethylation and OS was observed in NSCLC patients (pooled HR 1.74, 95% CI: 1.36–2.22) (Figure 2A). As for CRC, 9 studies involving 2752 patients are included in the analysis, indicative of the significant correlation between *p16* hypermethylation and OS (HR 1.80, 95% CI: 1.27–2.55) (Figure 2C). Moreover, the pooled HR estimate for OS of patients with NSCLC was 1.88 (95% CI: 1.30–2.73) in the analysis of the 2 studies with multivariate analysis, suggesting that *p16* hypermethylation might be an independent prognostic factor for patients with NSCLC.

We also performed subgroup analysis by region, publication year, REMARK score**,** ELCWP score**,** and number of patients. *p16* hypermethylation was significantly associated with OS of patients with NSCLC in Asia (HR 1.65, 1.16–2.35) and USA (HR 1.89, 1.27–2.82), and with OS of patients with CRC in Asia (HR 3.30, 1.68–6.46) and Europe (HR 1.49, 1.05–2.09) (Table 1). We also observed the significant correlation of *p16* hypermethylation and OS in studies with REMARK score greater than 12 in NSCLC (HR 1.79, 1.35–2.39) and CRC (HR 1.96, 1.16–3.34), further confirming the prognostic role of *p16* hypermethylation in these 2 cancer types (Figure 2A,C, and Table 1). Subgroup analysis by other factors including number of patients, publication year and ELCWP score did not alter the significant prognostic significance of *p16* hypermethylation regarding OS in NSCLC or CRC (Table 1).

**Table 1:** Meta-regression and subgroup analysis of the studies reporting the prognostic value of*p16* hypermethylation on OS of NSCLC and CRC.

Stratified analysis	No. of studies	No. of patients	Pooled HR(95%CI)		Meta-regression p-value	Heterogeneity	
			Fixed	Random		I^2^(%)	p-value
**NSCLC overall**	11	1654	1.60 [1.37, 1.86]	1.74 [1.36, 2.22]		53	0.01
**Region**					0.591		
Asia	7	904	1.60 [1.29, 1.99]	1.65 [1.16, 2.35]		55	0.03
USA	4	750	1.59 [1.28, 1.98]	1.89 [1.27, 2.82]		61	0.04
**Publication year**							
≤2005	5	639	1.45 [1.16, 1.80]	1.76 [1.11, 2.78]	0.790	68	0.008
>2005	6	1015	1.76 [1.42, 2.19]	1.77 [1.35, 2.33]		31	0.19
**REMARK score**					0.720		
≥12	4	727	1.77 [1.42, 2.22]	1.79 [1.35, 2.39]		37	0.16
<12	7	927	1.45 [1.17, 1.79]	1.75 [1.14, 2.68]		63	0.01
**ELCWP score(%)**					0.866		
<63	6	959	1.46 [1.18, 1.80]	1.79 [1.14, 2.82]		66	0.01
>63	5	695	1.76 [1.41, 2.20]	1.76 [1.32, 2.35]		37	0.14
**No. of patients**					0.270		
>150	5	1197	1.49 [1.24, 1.78]	1.55 [1.18, 2.03]		51	0.07
<150	6	457	1.93 [1.44, 2.58]	2.14 [1.34, 3.44]		55	0.04
**CRC overall**	9	2752	1.41 [1.21, 1.64]	1.80 [1.27, 2.55]		75	0.0001
**Region**					0.734		
Asia	4	393	3.32 [2.14, 5.15]	3.30 [1.68, 6.46]		56	0.08
Europe	3	902	1.52 [1.18, 1.96]	1.49 [1.05, 2.09]		35	0.21
USA	1	902	1.03 [0.81, 1.30]	1.03 [0.81, 1.30]			
Australia	1	555	1.40 [0.91, 2.16]	1.40 [0.91, 2.16]			
**Publication year**					0.395		
≤2005	4	815	1.93 [1.40, 2.65]	2.23 [1.25, 3.98]		61	0.05
>2005	5	1937	1.29 [1.09, 1.53]	1.56 [1.01, 2.44]		79	0.0007
**REMARK score**					0.722		
≥12	5	1933	1.37 [1.16, 1.62]	1.96 [1.16, 3.34]		86	0.00001
<12	4	819	1.55 [1.12, 2.16]	1.55 [1.12, 2.16]		0	0.48
**ELCWP score(%)**					0.211		
<63	4	807	2.05 [1.54, 2.72]	2.47 [1.32, 4.60]		64	0.04
>63	5	1945	1.22 [1.02, 1.45]	1.47 [1.08, 2.18]		72	0.007
**No. of patients**					0.107		
>150	4	2273	1.24 [1.06, 1.46]	1.29 [1.07, 1.71]		62	0.05
<150	5	479	2.81 [1.91, 4.13]	2.83 [1.57, 5.11]		57	0.06

Footnotes: NSCLC, non-small cell lung carcinoma; CRC, colorectal cancer; no., number; OS, overall survival.

**Figure 3:**
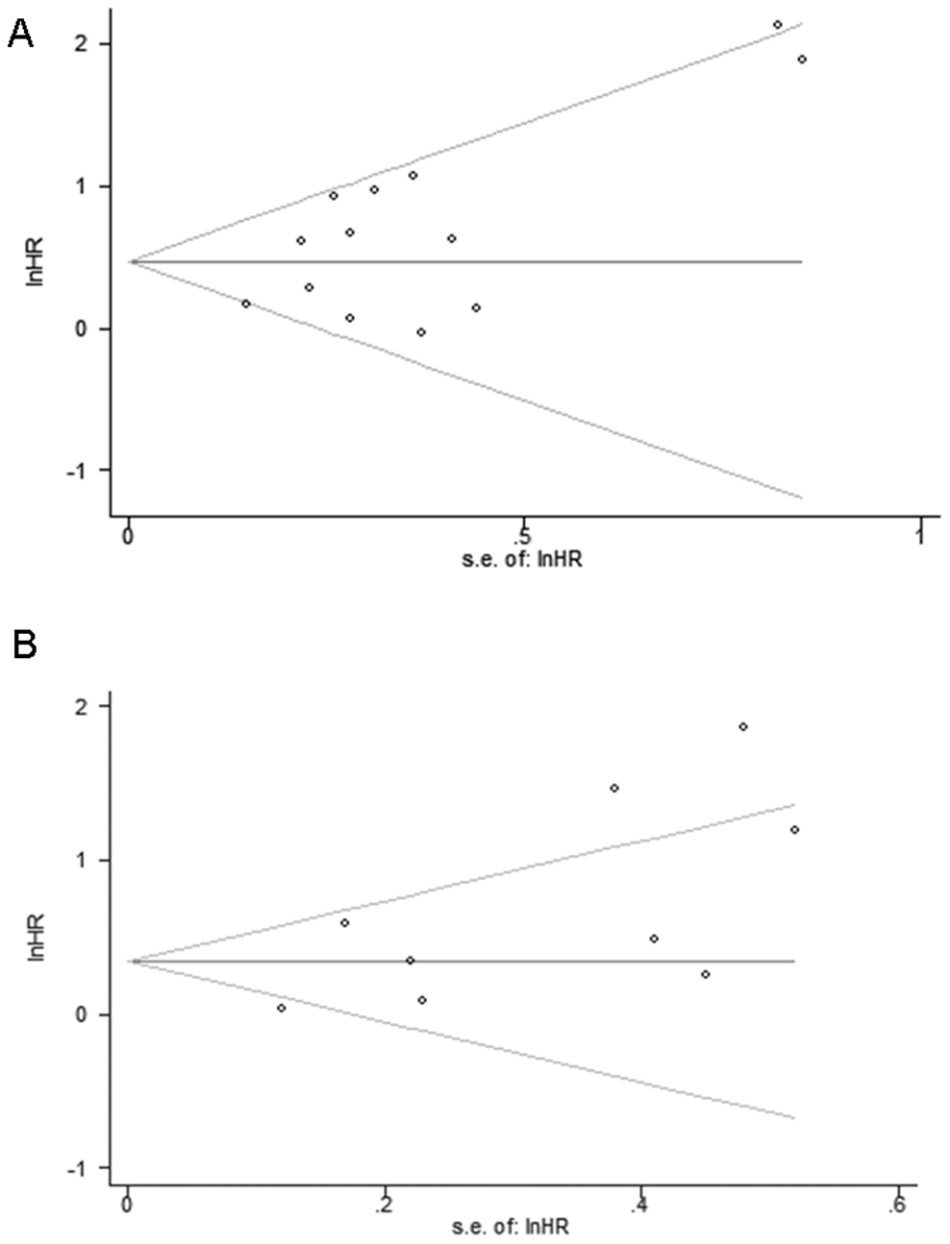
Funnel plots for the evaluation of potential publication bias in the impact of*p16* hypermethylation on OS of NSCLC (A) and CRC (B). The funnel graph plots the log of hazard ratio (HR) against the standard error of the log of the HR (an indicator of sample size). The circles indicate the individual studies in the meta-analysis. The line in the centre represents the pooled HR. The Egger test for publication bias was significant for OS of NSCLC (p = 0.042) and CRC (p = 0.033).

Owing to heterogeneity across studies reporting OS of patients with NSCLC (I^2^ = 53%) and CRC (I^2^ = 75%), we analyzed the source of the heterogeneity using meta-regression by the covariates including region, publication year, REMARK score, ELCWP score and number of patients. Whereas, meta-regression did not reveal that one of these covariates might account for part of the inter-study heterogeneity (Table 1). In addition, omitting a certain study did not reduce inter-study heterogeneity significantly in the sensitivity analysis. Assessment of publication bias revealed that the Egger test was significant regarding both NSCLC (p = 0.042) and CRC (p = 0.033), and the funnel plots for publication bias revealed a certain degree of asymmetry (Figure 3).

In addition, meta-analysis of 4 publications comprising 311 patients demonstrated the significant association of *p16* hypermethylation and DFS of NSCLC patients (HR 2.04, 95% CI: 1.19–3.50) in a random-effects model (Figure 2B). While the analysis on CRC did not demonstrate the significant correlation between *p16* hypermethylation and DFS of patients (HR 2.11, 95% CI: 0.75–5.95) in a random-effects model (Figure 2D). Overall test for heterogeneity recorded a I^2^ value of 52% (p = 0.08) for NSCLC and 85% (p = 0.001) for CRC, suggesting the presence of inter-study heterogeneity. Due to the limited number of studies on the analysis of *p16* hypermethylation and DFS of both tumor types, we did not perform the meta-regression. Of note, subgroup analysis also exhibited the significant association of *p16* hypermethylation and poor DFS of patients with NSCLC in studies with REMARK score greater than 12 (HR 2.12, 95% CI: 1.21–3.72) (Figure 2B). Sensitivity analysis for NSCLC and CRC revealed that heterogeneity was not generated by any single study.

### Association of*p16* Hypermethylation and Survival of Patients with Cancer Types Other than NSCLC and CRC

The meta-analysis was also performed for other cancer types on which more than 2 studies were eligible for inclusion. As indicated in table 2, the significant association was not found between *p16* hypermethylation and OS of esophageal cancer (HR 1.25, 95% CI: 0.38–4.13), gastric cancer (HR 4.64, 95% CI: 0.87–24.64), head and neck cancer (HR 0.96, 95% CI: 0.65–1.40), brain cancer (HR 1.33, 95% CI: 0.43–4.07), or bladder cancer (HR 1.77, 95% CI: 0.59–5.38). In addition, *p16* hypermethylation did not have significant correlation with DFS of hepatocellular cancer (HR 1.14, 95% CI: 0.67–1.95). While the meta-analysis indicated the significant association was present between *p16* hypermethylation and DFS of head and neck cancer (HR 2.24, 95% CI: 1.35–3.73) and the included 3 studies for the analysis all possessed REMARK score greater than 12. Due to the limited number of eligible studies (n = 2 to 3) and patients (n = 101 to 370) included in the analysis of cancer types other than NSCLC and CRC, we did not conduct the meta-regression and subgroup analysis for these carcinomas.

**Table 2:** Meta-analysis of effects of*p16* hypermethylation on OS and DFS of cancer types other than NSCLC and CRC.

Cancer type	No. of studies	No. of patients	Pooled HR(95%CI)		Heterogeneity	
			Fixed	Random	I^2^(%)	p-value
**OS**						
Esophageal cancer	2	202	1.01 [0.61, 1.68]	1.25 [0.38, 4.13]	81	0.006
Gastric cancer	2	160	2.69 [1.73, 4.16]	4.64 [0.87, 24.64]	70	0.07
Head and neck cancer	3	217	0.96 [0.65, 1.40]	0.96 [0.65, 1.40]	0	0.57
Brain cancer	2	370	1.48 [0.79, 2.78]	1.33 [0.43, 4.07]	66	0.09
Bladder cancer	2	129	1.77 [0.59, 5.38]	1.77 [0.59, 5.38]	0	0.74
**DFS**						
HCC	2	101	1.14 [0.67, 1.95]	1.14 [0.67, 1.95]	0	0.58
Head and neck cancer	3	229	2.24 [1.35, 3.73]	2.24 [1.35, 3.73]	0	0.77

Footnotes: NSCLC, non-small cell lung carcinoma; CRC, colorectal cancer; HCC, hepatocellular cancer; no., number;

OS, overall survival; DFS, disease-free survival.

## Discussion

Many efforts have been made to predict the prognosis of cancer patients posterior to surgical treatment by using the molecular analysis of the primary tumor and regional lymph nodes [3], [58]–[59]. Whereas, it remains a topic that needs exploration to assess the prognostic significance of molecular analysis comprising epigenetic markers in cancers, in order to better identify the proportion of patients who might need adjuvant therapy subsequent to surgical resection. Although numerous studies have been performed to assess the prognostic value of hypermethylation of the tumor suppressor *p16* promoter in different cancers, the results are still controversial and ambiguous.

To our knowledge, this meta-analysis is the first study to systematically assess the association between *p16* hypermethylation and prognosis of various cancer types. Interestingly, pooled analysis of the included studies exhibited a significant correlation between *p16* hypermethylation and poor OS of patients with NSCLC and CRC. Moreover, the pooled data of studies with multivariate analysis demonstrated the significant association between *p16* hypermethylation and OS of NSCLC, suggesting that *p16* hypermethylation might be an independent prognostic factor of poor survival in patients with NSCLC. Moreover, subgroup analysis indicated that *p16* hypermethylation was significantly associated with OS and DFS of NSCLC in the analysis of studies following REMARK guidelines more rigorously, further confirming the prognostic value of *p16* hypermethylation in NSCLC. *p16* hypermethylation also had an unfavorable impact on OS of CRC and DFS of head and neck cancer in studies following REMARK guidelines more rigorously. We did not find the significant association between *p16* hypermethylation and survival of other cancer types, possibly due to the limited number of studies identified for the analysis. More studies with a larger sample size on those cancer types are thus needed to further elucidate the prognostic value of *p16* hypermethylation in them.

In addition, the source of inter-study heterogeneity present in this analysis on OS of NSCLC and CRC was analyzed using meta-regression and subgroup analysis by region, publication year, REMARK score, ELCWP score and number of patients. The results did not show that one of these covariates was the source of heterogeneity. Furthermore, subgroup analysis by these factors did not alter the prognostic significance of *p16* hypermethylation regarding OS in these two cancer types. However, we cannot completely exclude the possibility that some of these covariates might potentially account for part of the heterogeneity, since the power of meta-regression analysis was known to be low. Owing to the limited number of studies included for cancer types other than NSCLC and CRC, meta-regression and subgroup analysis was not allowed to be conducted for them.

Although we observed the significant association between *p16* hypermethylation and poor long-term outcome of some types of cancer, the limitations of this meta-analysis cannot be ignored. The data of the studies included in the meta-analysis are not individual patient data which may provide more reliable detailed results as compared to data from published articles. Moreover, a certain degree of publication bias was found in the analysis concerning OS of NSCLC and CRC according to Egger test and funnel plot. The HR estimate in our meta-analysis might be overestimated because of publication and reporting bias. In addition, this meta-analysis was restricted to studies published in English language, owing to the fact that publications in other languages were usually not available for both authors and readers. Furthermore, studies with non-significant results are less often published or, if published, in a more brief way. Unpublished studies and conference abstracts were not included in our analysis, because the data that can be used for methodology assessment and meta-analysis were only available in full publications. As a result, the selection of publications might favor the trials possessing positive results which were more frequently published in English language. However, we made as complete search for literature as possible using the databases above mentioned, trying to minimize the publication and reporting bias.

To sum up, *p16* hypermethylation exhibited the significant association with OS of NSCLC and CRC, and with DFS of NSCLC and head and neck cancer. In addition, the results of the analysis on studies following REMARK guidelines more rigorously further confirm the predictive impact of *p16* hypermethylation for the prognosis of these cancer types, particularly NSCLC. Whereas, the large prospective clinical studies based on homogeneous series of patients are needed to further confirm the prognostic value of *p16* hypermethylation in different types of cancer.

## Supporting Information

Table S1 **Main characteristics of the included studies.**
(DOC)
