# Supplementary material for: The Prognostic Value of p16 Hypermethylation in Cancer: A Meta-Analysis
Source: PLoS One. 2013 Jun 21;8(6):e66587. doi: 10.1371/journal.pone.0066587 (PMC3689792; doi:10.1371/journal.pone.0066587)
Supplement: Table S1 — Main characteristics of the included studies. (DOC) [file pone.0066587.s001.doc]

Supplementary table 1 Main characteristics of the included studies

| First Author | Year | Region  (country) | Tumor type | Stage of disease | Number of patients | Methylation rate(%) | Methylation detection method | Follow Up (months) | | Outcomes  reported | Multivariate analysis |
| --- | --- | --- | --- | --- | --- | --- | --- | --- | --- | --- | --- |
|  |  |  |  |  |  |  |  | median | range |  |  |
| Barault | 2008 | France | CRC | I-IV | 498 | na | MSP | N/A | N/A | OS | Yes |
| Esteller | 2001 | Spain | CRC | Dukes A-C | 86 | 35 | MSP | 68 | 16-89 | OS | no |
| Ishiguro | 2006 | Japan | CRC | I-IV | 88 | 23 | MSP | 53.2 | N/A | OS | no |
| Kim | 2010 | Korea | CRC | N/A | 131 | 11 | MSP | 49 | 1-116 | OS, DFS | no |
| Liang | 1999 | China | CRC | Dukes B_2_ | 84 | 29 | MSP | N/A | 60-86 | OS | no |
| Maeda | 2003 | Japan | CRC | Dukes B-C | 90 | 13 | qMSP | 54.5 | N/A | OS | no |
| Mitomi | 2010 | Japan | CRC | II-IV | 151 | 30 | qMSP | 79 | 60-123 | DFS | no |
| Shima | 2011 | USA | CRC | I-IV | 902 | 30 | qMSP | 146 | N/A | OS | yes |
| Veganzones-de-Castro | 2012 | Spain | CRC | Dukes A-D | 318 | 25 | qMSP | 92 | 75-111 | OS, DFS | no |
| Ward | 2003 | Australia | CRC | I-IV | 555 | 23 | MSP | 32 | 1-60 | OS | no |
| Buckingham | 2010 | USA | NSCLC | I-II | 125 | 21 | qMSP | N/A | 12-144 | OS | no |
| Chen | 2002 | China | NSCLC | I-IV | 67 | 57 | MSP | 22.4 | 0.5-84 | OS | no |
| Gonzalez-Quevedo | 2004 | Spain | NSCLC | I-IV | 47 | 36 | MSP | 37.6 | 11-79 | DFS | no |
| Gu | 2006 | USA | NSCLC | I-III | 155 | 22 | qMSP | N/A | N/A | OS | yes |
| Kurakawa | 2001 | Japan | NSCLC | I-IV | 45 | 38 | MSP | N/A | N/A | OS | no |
| Nakata | 2006 | Japan | NSCLC | I-IV | 224 | 22 | MSP | 60. 8 | 20.2-127.0 | OS | no |
| OTA | 2006 | Japan | NSCLC | I-IV | 238 | 15 | qMSP | 45 | 2-149 | OS | no |
| Suzuki | 2010 | Japan | NSCLC | I-IV | 229 | 31 | MSP | N/A | N/A | OS | no |
| Tanaka | 2005 | Japan | Lung adenocarcinoma | I-III | 57 | 40 | MSP | N/A | N/A | OS | no |
| Toyooka | 2004 | USA | NSCLC | I-III | 351 | 25 | MSP | N/A | N/A | OS | no (ADC+SCC)  yes (ADC alone) |
| Wang | 2004 | USA | NSCLC | I-IIIA | 119 | 49 | MSP | 51 | 16-130 | OS, DFS | no |
| Yanagawa | 2007 | Japan | NSCLC | I-III | 101 | 27 | MSP | 43.2 | 4.8-76.9 | DFS | no |
| Yoshino | 2009 | Japan | NSCLC | IA | 44 | 25 | MSP | 77.4 | 16.0-147.2 | OS, DFS | no |
| Li | 2004 | Japan | HCC | I-IV | 50 | 58 | MSP | 46 | 6-71 | DFS | no |
| Li | 2010 | China | HCC | I-III | 51 | 78 | MSP | 31 | 2-118 | OS, DFS | no |
| Alaa | 2009 | Japan | Esophageal cancer | I-IV | 82 | 33 | MSP | N/A | N/A | OS | yes |
| Lu | 2011 | China | Esophageal cancer | I-IV | 120 | 88 | MSP | 42 | 0.36-55.9 | OS | no |
| Shi | 2012 | China | Gastric cancer | I-IV | 119 | 45 | MSP | N/A | N/A | OS | no |
| Mitsuno | 2007 | Japan | Gastric cancer | I-IV | 56 | 27 | MSP | 32.3 | 6.0-78.5 | DFS | no |
| Ben | 2011 | Tunisia | Gastric cancer | I-IV | 41 | 32 | MSP | N/A | 0.03-104.6 | OS | yes |
| Diskshit | 2007 | Europe | Head and neck cancer | I-IV | 126 | 44 | MSP | 97.2 | 0-252 | OS | no |
| Dong | 2012 | China | Head and neck cancer | I-IV | 30 | 50 | MSP | 38.03 | 8-60 | OS | yes |
| Sailasree | 2008 | India | Head and neck cancer | I-IV | 116 | 29 | MSP | 23 | 0-50 | DFS | no |
| Su | 2010 | China | Head and neck cancer | I-IV | 52 | 29 | qMSP | N/A | N/A | DFS | no |
| Taioli | 2009 | USA | Head and neck cancer | I-IV | 61 | 11 | qMSP | N/A | 16.9-114.7 | OS, DFS | yes |
| Kawamoto | 2006 | Japan | Bladder cancer | I-IV | 45 | 18 | qMSP | 34.3 | 27.0-100.1 | OS | no |
| Yurakh | 2006 | Spain | Bladder cancer | I-IV | 84 | 6 | qMSP | 36.4 | 1.3-49.2 | OS, DFS | no |
| Sharma | 2009 | India | Breast cancer | I-III | 101 | 50 | MSP | N/A | N/A | OS, DFS | yes |
| Staume | 2002 | Sweden | Melanoma | N/A | 59 | 19 | MSP | 76 | 13-210 | OS | no |
| Feng | 2012 | USA | Brain cancer | N/A | 321 | 70 | qMSP | N/A | N/A | OS | yes |
| Kuo | 2009 | China | Brain cancer | II-III | 49 | 47 | MSP | 83 | 3-204 | OS | no |
| Kawaguchi | 2003 | Japan | Leiomyosarcoma | I-IV | 37 | 22 | MSP | 58.4 | 14-258 | OS | no |
| Katsaros | 2004 | Italy | Ovarian cancer | I-IV | 212 | 40 | MSP | 31 | 0.6-114 | OS | no |
| Viadaurreta | 2008 | Spain | Renal cancer | I-IV | 48 | 23 | MSP | 76 | 2-117 | OS, DFS | no |
| Guerrero | 2008 | Spain | Penile cancer | I-IV | 24 | 38 | MSP | 72 | 5-228 | OS, DFS | no |

Footnotes: NSCLC, non-small cell lung carcinoma; CRC, colorectal cancer; HCC, hepatocellular cancer; N/A, not available; OS, overall survival; DFS, disease-free survival; MSP, methylation specific PCR; qMSP, quantitative MSP.
